# Adverse events in prehospital emergency care: a trigger tool study

**DOI:** 10.1186/s12873-019-0228-3

**Published:** 2019-01-24

**Authors:** Magnus Andersson Hagiwara, Carl Magnusson, Johan Herlitz, Elin Seffel, Christer Axelsson, Monica Munters, Anneli Strömsöe, Lena Nilsson

**Affiliations:** 1 Faculty of Caring Science, Work Life and Social Welfare, University of Borås, SE-501 90 Borås, Sweden; 2 Department of Molecular and Clinical Medicine, University of Gothenburg and Sahlgrenska University Hospital, SE-405 30 Gothenburg, Sweden; 3 Department of Ambulance Care, Södra Älvsborg Hospital (SÄS), SE-501 82 Borås, Sweden; 4 Department of Ambulance Care, Region of Dalarna, SE-791 29 Falun, Sweden; 5 School of Health, Care and Social Welfare, Mälardalens högskola, SE-721 23 Västerås, Sweden; 6 Department of Anaesthesiology and Intensive Care and Department of Medical and Health Sciences, Linköping University, SE-581 85 Linköping, Sweden

**Keywords:** Emergency medical service, Adverse events, Patient safety, Trigger tool, Prehospital

## Abstract

**Background:**

Prehospital emergency care has developed rapidly during the past decades. The care is given in a complex context which makes prehospital care a potential high-risk activity when it comes to patient safety. Patient safety in the prehospital setting has been only sparsely investigated. The aims of the present study were 1) To investigate the incidence of adverse events (AEs) in prehospital care and 2) To investigate the factors contributing to AEs in prehospital care.

**Methods:**

We used a retrospective study design where 30 randomly selected prehospital medical records were screened for AEs each month in three prehospital organizations in Sweden during a period of one year. A total of 1080 prehospital medical records were included. The record review was based on the use of 11 screening criteria.

**Results:**

The reviewers identified 46 AEs in 46 of 1080 (4.3%) prehospital medical records. Of the 46 AEs, 43 were classified as potential for harm (AE1) (4.0, 95% CI = 2.9–5.4) and three as harm identified (AE2) (0.3, 95% CI = 0.1–0.9). However, among patients with a life-threatening condition (priority 1), the risk of AE was higher (16.5%). The most common factors contributing to AEs were deviations from standard of care and missing, incomplete, or unclear documentation. The most common cause of AEs was the result of action(s) or inaction(s) by the emergency medical service (EMS) crew.

**Conclusions:**

There were 4.3 AEs per 100 ambulance missions in Swedish prehospital care. The majority of AEs originated from deviations from standard of care and incomplete documentation. There was an increase in the risk of AE among patients who the EMS team assessed as having a life-threatening condition. Most AEs were possible to avoid.

**Electronic supplementary material:**

The online version of this article (10.1186/s12873-019-0228-3) contains supplementary material, which is available to authorized users.

## Background

Patient safety among those who require care within a hospital environment has attracted a great deal of attention following the Institute of Medicines’ report, *To err is human,* in 2000 [1]. Even though the incidence of adverse events in hospital settings is relatively well known [2], hospital patient safety research struggles to find methods for identifying adverse events and prioritizing effective patient safety interventions [3, 4].

The patient safety in the prehospital setting is even less well documented. A report from the National Patient Safety Foundation [5] pointed at the slow progress in patient safety research and the lack of research outside the hospital setting. Thus the knowledge gap is huge.

A number of factors highlight the urgent need for further exploring how the assessment and care that is offered by health care providers before arrival in hospital influence patient safety among those who call for Emergency Medical Service (EMS) (see Additional file 1: Table S1 for examples). This is partly explained by the fact that during the past decades, with differences between organizations, prehospital care has been transformed from a transport organization to an integrated part of the health-care system [6]. The rapid transition of prehospital care poses great challenges for the involved organizations in terms of education, equipment, methods and decision support. In addition to advanced care for critically ill or injured patients [7], the EMS clinicians make decisions on the level of care for other patients; to stay at home with self-care advice [8], to be transported to primary care [9], to be transported to the nearest emergency department (ED) or to bypassing the ED to be transported directly to a specialist assessment or treatment within the hospital [10]. Examples of the latter group are patients who suffer from stroke and myocardial infarction. A limited recognition of these diseases in the prehospital setting may delay the time to reperfusion and thereby increase the risk of extensive damage to the brain or the myocardium.

Prehospital care is a potentially high-risk activity. The EMS clinicians assess patients of all ages with medical conditions of all kinds and sometimes with a limited capability to communicate. Care is given in sometimes difficult environments, 24 h a day and generally far from medical support [11, 12]. The complex context poses many barriers to effective research [13]. The prehospital organizations also have problems identifying, reporting and disclosing adverse events [14, 15].

Based on this background, the goal of the present study was to make a contribution to prehospital patient safety research with the following aims:

1. To investigate the incidence of adverse events in prehospital care.
2. To investigate the factors contributing to adverse events in prehospital care.

## Method

### Design

We used a retrospective study design of prehospital medical records from three prehospital organizations in Sweden between 1 Jan and 31 Dec 2016. As a result, 30 randomly selected prehospital medical records were screened for adverse events each month in each of the prehospital organizations. Eleven screening criteria were used for the medical record review [16]. These criteria were:

1. Missing, incomplete, or unclear documentation for the following: chief complaint, physical assessment, vital signs, hemodynamic monitoring (e.g., ETC02), allergies, pertinent history or medications, patient condition at handoff of facility
2. Time from initial patient contact to transfer of care exceeds accepted standards
3. Injury to patient or team member during patient encounter/transport (e.g., stretcher drop, needle stick, or other)
4. Request for additional resources, personnel, or supervisor due to change in patient condition
5. A worsening trend (deterioration) in patient hemodynamic or mental status indicators (e.g., vital signs, level of consciousness Glasgow Coma Scale score)
6. Cardiac arrest during transport
7. Use of any of the following interventions: cardioversion, defibrillation, transcutaneous pacing, advanced airway attempt, surgical airway, intraosseous access, chest decompression, chest tube
8. Failure of any intervention or procedure during patient care (some examples include: inability to obtain vascular access after a reasonable amount of time or number of attempts, failed intraosseous access, failed nasogastric tube placement, failed Foley placement, failed cardioversion, failed defibrillation, failed transcutaneous pacing, failed advanced airway or rescue airway, failed surgical airway, failed chest decompression)
9. Use of following medications or fluids: (blood products, vasopressors or inotropes [e.g., dobutamine, dopamine], naloxone, rapid sequence intubation medications [e.g., succinylcholine])
10. Evidence suggestive of deviation from standard of care by performing an intervention or administering a medication that appears to be outside protocol or failure to perform an intervention or provide a medication that is within the standard of care
11. Medication error (e.g., administering wrong or unapproved dose, administering wrong or unapproved medication, administering medication via wrong or unapproved route)

### Settings

The study was conducted in three prehospital organizations in Sweden during a period of one year. The three prehospital organizations were chosen to represent three different types of organization; one urban environment, with the majority of the ambulance missions having generally short transportation times. The second organization represented a mixed area, with both rural and urban missions, thereby including both short and long mission times, and the last organization represented a more rural area with longer mission times as compared with the other two. The prehospital care was mainly based on ground-based EMS units. There were also two ambulance helicopters in the organizations which were operated by a physician and a nurse. Prehospital care given by physicians was not included in this study. The included ambulance organizations constituted a reasonable representation of prehospital care in Sweden (see Table 1 for organizational differences).

**Table 1:** Demographic information on the three included emergency medical service organizations

	Urban organization	Mixed organization	Rural organization
Area	900 km2	6464 km2	28,030 km2
Population	660,000	302,441	284,531
Population/km2	733	47	10
EMS missions/year	82,419	39,517	37,149
Mission time (minutes) (median)	47	62	78
Proportion of RN	88%	96%	89%

The data are based on the organizations’ reports from 2016

*EMS* emergency medical service, *RN* registered nurse

### Population

The ambulance personnel in the three participating organizations included registered nurses (RN), with and without specialist education in prehospital care, and emergency medical technicians (EMT). Prehospital care in Sweden is divided into regions and the Swedish National Board of Health and Welfare allows the respective region to a large extent to plan and execute prehospital care according to its own needs [17]. In the past, ambulance care was executed by EMTs with a short education in prehospital care (20–40 weeks), but, since 2005, all ambulances are staffed by at least one RN with medical responsibility, in accordance with Swedish law [18]. In Sweden, RNs have three years’ education ending up with a bachelor’s degree. At the present time, there are no requirements for a specialist nurse in prehospital emergency care (with one year of additional training). An ambulance team in Sweden may consist of the combinations of RN, prehospital emergency care nurse, RN with another specialist education such as anaesthesia or intensive care and EMT [19]. The RN independently administers around 30 different drugs according to written guidelines and general delegation. The proportion of RNs in Swedish prehospital care has been estimated at 68–78%. The proportion of RNs with specialist education varies widely between regions from 20 to 85% [20]. RNs in prehospital care in Sweden are a fairly stable group. For example, 42% of the RNs in the study population had worked for more than ten years in prehospital care. In this study, we use the expression “EMS clinicians” for all types of prehospital staff.

### Inclusion and exclusion criteria

The inclusion criterion was a ground based ambulance mission which included patient assessment and care.

The exclusion criteria were:

1. Patient < 18 years
2. Ambulance missions without patient contact
3. Ambulance missions which represented support to another ambulance team
4. Ambulance transportation between health-care facilities
5. Patient deceased upon EMS arrival

The reason for excluding children was that patient safety issues among paediatric patients are unique [21] and experience with other trigger tools has revealed that paediatric populations frequently require different triggers [22].

### Materials

The sample comprised 30 prehospital records each month based on recommendations from the Institute for Healthcare Improvement [23]. The 30 records were selected by a random number generator. The records were first screened for inclusion and exclusion criteria. In cases in which the records were excluded, the randomization procedure was repeated until 30 records which fulfilled the inclusion criterion and without exclusion criteria were sampled. A total of 1080 ambulance missions were included in the study.

Demographic data (age and gender) on the patients were noted, together with the discharge diagnosis, time of year and emergency priority level of the mission; priority 1 – life-threatening conditions; priority 2 – urgent but not life-threatening conditions; priority 3 – neither life-threatening nor urgent conditions.

When an adverse event was detected in the prehospital record, the relevant hospital record was reviewed to determine whether the adverse event was an adverse event 1 – potential for harm or an adverse event 2 – harm identified. The definition of an adverse event was “An adverse event in the EMS is a harmful or potentially harmful event occurring during the continuum of EMS care that is potentially preventable and thus independent of the progression of the patient’s condition” [24].

The screening instrument was a trigger tool originally designed for helicopter-based emergency care developed by Patterson et al. [16]. We applied this trigger tool to 30 prehospital records in a pilot test. Based on this experience, it was agreed that the instrument could also be suitable for ground-based prehospital care. The trigger tool was translated into Swedish by a professional translator with English as his/her native language. No other adjustments to the trigger tool were made.

Each participating organization had one responsible primary reviewer (*N* = 3). These reviewers were all active ambulance nurses working in the organization with knowledge of the particular organization’s routines, processes, medical record systems and guidelines. In addition to the three primary reviewers, the review team consisted of two researchers/ prehospital emergency care nurse, one cardiologist and one anaesthesiologist. The three primary reviewers had one initial rater-training session that lasted for three hours. The complete review team had four sessions during the study period. During the meetings, experiences were exchanged between the reviewers and difficult medical records were discussed and resolved by consensus in the team. Outside the four official meetings, the three primary reviewers had continuous contact during the study period via mail and telephone. All medical records which included any adverse event (adverse event 1 or adverse event 2) were screened and discussed in the complete review team and the final decision was made through consensus meetings during the study period.

To assess inter-rater reliability, the three primary reviewers independently reviewed the same thirty records from one month. The review took place in their respective home offices without any communication during the review.

The prehospital record screening process was conducted in three steps. In the first step, the reviewer used a list of 11 triggers. A trigger is not by definition an adverse event. The function of the trigger is to alert the rater to something that might point to an adverse event. If any triggers were found in the first step, they were classified in five different categories in the next step. Category 1: The trigger was the result of action(s) by the patient. Category 2: The trigger was the result of action(s) or inaction(s) by the crew. Category 3: Failure of the equipment, failure to troubleshoot and correct common problems with the equipment, or failure to remove defective equipment from service. Category 4: Factors that may result from weather conditions or factors on the ground/at the scene (or other). Category 5: The proximal cause of the trigger (regardless of severity) cannot be determined by the information available in the chart. In the last step, the trigger was categorized by severity; adverse events 1 = adverse events present – potential for harm but there is no evidence that any harm occurred or adverse event 2 = adverse event present – harm was identified regardless of severity. One or more triggers can point to the same adverse event.

More information on the study can be found in a previously published study protocol [25].

### Statistical analysis

All the data were recorded in a database designed for this project. Data are presented as the mean (standard deviation (SD)), ratio, frequencies (percent, 95% confidence interval [CI])) or unadjusted odds ratios (OR, 95% CI). We used cross-tabulation to assess the distribution of the trigger number and category number of the independent variables with the dependent variables adverse event 1 and adverse event 2, as well as to assess the distribution of the independent variables of gender, age, discharge diagnosis group, time of year, EMS organization and priority levels with the dependent variable adverse event (adverse event 1 and adverse event 2 combined). We constructed two univariate logistic regression models with the dependent variable adverse event and the independent variables triggers 1–11 and categories 1–5. For the more exploratory variables of gender, age, discharge diagnosis group, time of year, EMS organization and EMS priority, a univariate logistic regression model was constructed for each variable and significant variables (*p* < 0.05) were then included in a multivariate model with the forward conditional method. The model calibration analysis was carried out with Hosmer-Lemeshow statistics. A *P* value of < 0.05 was regarded as statistically significant.

The degree of agreement between reviewers on whether or not the medical records indicated an adverse event was determined by Light’s kappa [26]. Kappa was computed for each coder pair and then averaged to provide a single index of inter-rater rating, IRR.

For all statistical analyses and data processing, the SAS package, version 9.1, and the SPSS 21.0 statistical software program (SPSS Inc., Chicago, IL) were used.

## Results

**Fig. 1:**
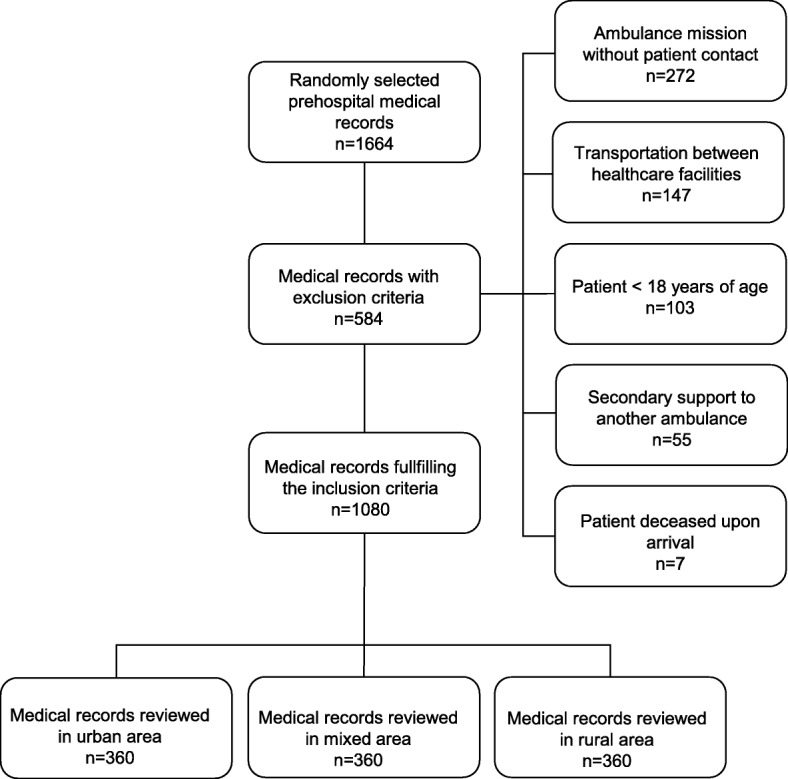
Flow chart showing the inclusion process of prehospital records for review

During the study period, the prehospital organizations had 159,085 ambulance missions. Of them, 107,968 were primary missions that included patient contact and patient assessments. Of the primary missions, 78% of the patients were transported to a health-care facility and the rest (22%) stayed on the scene with self-care advice or were referred to primary care. During the sampling process, 1664 prehospital medical records were collected. Of them, 584 (35%) medical records were excluded because of exclusion criteria (Fig. 1). The reviewers gathered complete data from 1080 prehospital medical records and, for those transported to hospital, the associated hospital medical records. Among the included patients, 49% were women. The patients’ mean age was 64 years (SD = 22). The mean of the total prehospital time (from dispatch to hand-over) was 57 min (SD = 25), the mean time from dispatch to first patient contact was 16 min (SD = 9) and the mean on-scene time was 24 min (SD = 17). The inter-rater reliability analysis between the reviewers showed к = 0.74 (good agreement).

### Potential for harm (adverse event 1) and harm identified (adverse event 2)

The reviewers identified 46 medical records with adverse events. Only one adverse event was detected per affected patient. These patients constituted 4.3% of the total sample. Of the 46 adverse events, 43 were classified as potential for harm (adverse event 1) (4.0, 95% CI = 2.9–5.4) and three as harm identified (adverse event 2) (0.3, 95% CI = 0.1–0.9).

### Trigger origin for cases with adverse events

The two most used triggers which identified an adverse event were 1) T10; *Evidence suggestive of deviation from standard of care by performing an intervention or administering a medication that appears to be outside protocol, or failure to perform an intervention or provide a medication that is within the standard of care* and 2) T1: *Missing, incomplete, or unclear documentation.* Two triggers were associated with a significant increased risk of adverse events: T2; *Time from initial patient contact to transfer of care exceeds accepted standards*, with an OR of 7.9 (95% CI = 3.0–20.1), and T10; with an OR of 20.8 (95% CI = 9.3–55.9). In Table 2, the association between each trigger and the risk of an adverse event is described for both adverse event 1 and adverse event 2 (Table 2).

**Table 2:** Trigger origin for cases containing adverse events (AE)

Trigger	Triggers (n)	Triggers related to potential for harm (AE 1) (n (% (95% CI))	Triggers related to harm identified (AE 2) (n (% (95% CI))
T1: Missing, incomplete, or unclear documentation	652	35 (5.4 (3.9–7.6))	3 (0.5 (0.1–1.5))
T2: Time from initial patient contact to transfer of care exceeds accepted standards.	47	8 (17.0 (8.1–31.3))	1 (2.1 (0.1–12.7))
T3: Injury to patient or team member during patient encounter/transport	1	0 (0 (0–0))	0 (0 (0–0))
T4: Request for additional resources, personnel, or supervisor due to change in patient condition	3	0 (0 (0–0))	0 (0 (0–0))
T5: A worsening trend in patient hemodynamic or mental status indicators	7	1 (14.3 (0.7–58.0))	1 (14.3 (0.7–58.0))
T6: Cardiac arrest during transport	4	0 (0 (0–0))	1 (25.0 (1.3–78.1))
T7: Use of any of the following interventions: cardioversion, defibrillation, transcutaneous pacing, advanced airway attempt, surgical airway, intraosseous access, chest decompression, chest tube	7	1 (14.3 (0.7–58.0))	0 (0 (0–0))
T8: Failure of any intervention or procedure during patient care	9	2 (22.2 (3.9–59.8))	1 (11.1 (0.6–49.3))
T9: Use of following medications or fluids: blood products, vasopressors or inotropes, naloxone	10	1 (10 (0.5–45.9))	0 (0 (0–0))
T 10: Evidence suggestive of deviation from standard of care by performing an intervention or administering a medication that appears to be outside protocol or failure to perform an intervention or provide a medication that is within the standard of care	305	37 (12.1 (8.8–16.6))	3 (1.0 (0.3–3.1))
T 11: Medication error	24	4 (16.7 (5.5–38.2))	1 (4.2 (0.2–23.1))

One AE can be connected to several triggers

*AE* adverse event

*CI* confidence interval

### Possible cause of adverse events

Category 2: *The adverse event was the result of action(s) or inaction(s) by the crew* was the most frequent cause of an adverse event (Table 3). A univariate logistic regression showed that the category with the highest risk of an adverse event was Category 3: *Failure of the equipment, failure to troubleshoot and correct common problems with the equipment,* with an OR of 49.7 (95% CI = 5.8–359.8). The second highest odds for an adverse event was Category 2 with an OR of 4.8 (95% CI = 2.2–12.8). In Table 3, the association between patients with a trigger within each of the five categories of possible underlying causes and the risk of an adverse event with 95% CI is described for both adverse event 1 and adverse event 2.

**Table 3:** Categorization of adverse events

Category	Patients (n)	Triggers (n)	Patients related to potential for harm (AE 1) (n (% (95% CI))	Patients related to harm identified (AE 2) (n (% (95% CI))
Category 1: The AE was the result of action(s) by the patient.	68	102	3 (4.4 (1.1–13.2))	0 (0 (0–0))
Category 2: The AE was the result of action(s) or inaction(s) by the crew.	622	898	36 (5.8 (4.1–8.0))	2 (0.3 (0.1–1.3))
Category 3: Failure of the equipment, failure to troubleshoot and correct common problems with the equipment	5	5	2 (40.0 (7.3–83.0))	0 (0 (0–0))
Category 4: Factors that may result from weather conditions or factors on the ground/at the scene	0	0	0 (0 (0–0))	0 (0 (0–0))
Category 5: The cause of the AE cannot be determined by the information available in the chart.	40	64	2 (5.0 (0.9–18.2))	1 (2.5 (0.1–14.7))

*AE* adverse event

*CI* confidence interval

### Gender, age, discharge diagnosis, time of year, EMS organization and priority level

A multivariate logistic regression analysis including the significant variables, EMS organization and priority during EMS transport, in univariate models as independent variables, shows that there was an significant increase in OR for adverse events (adverse event 1 and adverse event 2 combined) among patients classified as priority 1 during EMS transport (OR = 6.40; 95% Cl = 2.10–19.46). The absolute risk for adverse events among patients classified as priority 1 was 16.5%. There was also an increase in OR for adverse events in the mixed EMS organization (OR = 8.98; 95% Cl = 2.68–30.11) (Table 4).

**Table 4:** Frequencies of adverse events in different categories (potential for harm (AE1) and harm identified (AE2) combined)

Categories	Patients		Adverse events
	N (1,080)	%	/100 missions
Gender			
Female	528	48.9	3.4
Male	546	50.6	4.9
Missing information	6		
Age			
18–39	203	18.8	1.5
40–69	323	29.9	5.6
70 +	554	51.3	4.5
Discharge diagnosis group			
Traumatic injury	138	12.8	4.3
Neurological	134	12.4	3,0
Cardiovascular	134	12.4	8.2
Gastrointestinal and urinary tract	104	9.6	2.9
Infections	96	8.9	7.3
Respiratory	46	4.3	2.2
Psychiatric	43	4.0	2.3
Endocrine system	14	1.3	14.3
Intoxication	11	1.0	9.1
Gynecological	6	0.6	0
Other diagnoses	87	8.1	4.6
Patient left at home or transported to primary care	267	24.7	2.2
Time of year			
January–April	360	33.3	2.2
May–August	360	33.3	4.8
September–December	360	33.3	5.8
EMS organization			
Urban	360	33.3	3.3
Mixed	360	33.3	8.5^a^
Rural	360	33.3	0.9
Priority to hospital			
Priority 1	127	11.8	16.5^a^
Priority 2	550	50.9	2.4
Priority 3	168	15.6	4.8
No transport by EMS	206	19.1	1.9
Missing information	28	2.6	

^a^Significant odds ratio

*EMS* emergency medical service

## Discussion

In comparison to hospital trigger tool studies, the present study revealed lower frequencies of adverse events (4.3%). The latest Swedish review of hospital patient safety reported an adverse event rate of 14% [26]. Several factors might contribute to the finding that prehospital care appears to have a lower risk rate. The prehospital care phase is much shorter in terms of time and there is one clinician who can give the patient their full attention during this period. In hospital care, the care event can last for weeks with many handovers with the risk of communication errors, potentially dangerous surgical interventions and medication treatments and the risk of hospital-related infections. The patients in prehospital care have varied levels of care needs. It has been estimated that 16 to 52% of EMS missions are unnecessary [27]. This contextual difference might contribute to the marked difference from the previously reported adverse event prevalence. In connection with the developing process of a prehospital trigger tool, Howard et al. [28] found an adverse event rate of 0.6/100 patient encounters among a sample of 159 prehospital medical records from an ambulance organization in Qatar. The sample only included low-risk/high-frequency cases. Meckler et al. [29] included 378 prehospital records in a chart review of the most severely ill or injured paediatric patients. They found unintended injuries, near misses, suboptimal actions, errors, or management complications in 70% of the charts in that population. In an agreement test where EMS medical director physicians reviewed 250 prehospital medical records from a convenience sample, Patterson et al. [24] found adverse events in 48% of the records.

These studies are difficult to compare with the present study. The main reason is the study sample. The former studies used directed sampling with paediatric patients, patients with severe conditions or low-risk patients. The main conclusion from these previous studies is that adverse events appeared to be more common among patients with severe conditions. This is confirmed by a study which reviewed reports from an anonymous prehospital reporting system. In the system, 82% of the reports describe incidents in patients with life-threatening or potentially life-threatening situations [30]. Moreover, in the present study, patients classified as priority level 1 (life threatening) by the EMS clinicians had a higher risk of adverse events compared with lower priority levels.

### Adherence to guidelines

There can be several reasons for deviations from guidelines. Previous prehospital patient safety studies have found that bias in clinical reasoning and decision-making is a large-scale risk for prehospital patient safety [6, 31–34]. The reason for this could be the rapid development of prehospital care which imposes heavy demands on the prehospital assessment. There is a risk that prehospital organizations have not adapted to this rapid development in educational issues, for example.

In addition to educational reasons, the lack of appropriate tools to support prehospital clinicians is a possible reason for the problem with deviation from standards [32]. Another identified issue is the lack of compliance with protocols and guidelines [35–39]. In a systematic review, Ebben et al. [40] found that compliance with prehospital guidelines varied from 7.8 to 95%. The lowest compliance was seen in cardiology treatment recommendations related to myocardial infarction and cardiac arrest and the highest compliance was found in connection with treatment recommendations related to oxygen administration and septicaemia and to monitoring recommendations related to oxygen administration. There are several possible reasons for poor compliance. EMS clinicians are dependent on guidelines and protocols [11, 12] but paper-based format of the tools has been an obstacle to using them explicitly in the patient assessment. Studies have showed that compliance increases when the same guidelines and protocols are integrated in a computerized decision-support system [41, 42]. Another reason for a poor compliance rate could be the lack of control from the prehospital organization’s perspective, since in some organizations, structured audit of the prehospital records is not conducted [11].

### Missing, incomplete, or unclear documentation

The quality of documentation in the prehospital medical records has an effect on patient safety. There are some previous indications that prehospital documentation is problematic. For example, missing physiological data at the scene in connection with trauma have been associated with increased mortality [43]. One study [44] found that up to 30% of critical information in connection with the handover of trauma patients was lost. This also highlights the need for high-quality prehospital medical records. Another study [45] found that, in most cases, the prehospital medical records were unavailable when critical medical decisions were made at the ED. The reason was that the EMS clinicians first used a paper-based documentation system in the field and, after handover at the ED, they produced the final version in a digital system. Poorly designed systems can foster errors instead of reducing them [46].

### Adverse events in prehospital care

To be able to strengthen the prehospital system, more knowledge of the nature of adverse events in prehospital care is needed. More research on prehospital clinical reasoning and decision-making is also important. This knowledge is crucial for the development of future prehospital information technology. We need more knowledge of the effect of different education strategies and the impact on patient safety. There is also a need to strengthen user involvement when developing new technology and new methods for prehospital care. One way of accomplishing user involvement is to use highly contextual simulation laboratories in the development and implementation process [47, 48]. The differences between different EMS organizations found in this study have to be further investigated.

### Limitations

The present study has limitations. The risk of disagreement between reviewers can lead to bias in the use of trigger tools [49]. Classen et al. [50] recommend a two-stage review process in which primary reviewers conduct the first review, followed by two physicians who determine the final results in consensus. Patterson et al. [51] found that group-based consensus had greater reliability in comparison with an independent reviewer system. In the present study, a mixture of independent reviews and group-based reviews has been used to increase the reliability of the study. Another method for strengthening study design is reviewer training and tests for inter-rater reliability.

One potential source of bias was that the primary reviewers reviewed the reports from their own organization. There is a risk that they could minimize the identification of adverse events to protect the organization and colleagues. To minimize this bias, we performed an IRR measurement and re-reviewed 10% of the medical records in a group of two other ambulance nurses. Unclear records were further reviewed by two physicians. In our opinion, it was important that the primary reviewer had experience of organizational routines and guidelines.

Another potential source of bias is the use of medical records as a data source. As in all retrospective record review studies, adverse events that were not documented in the medical records could not be detected. The incidence of adverse events is probably underestimated compared with direct observation [52]. The risk could be potentially even more extensive when it comes to prehospital care, as, on many occasions, the medical records are completed a long time after the first patient contact [45] and there are known quality problems with prehospital documentation [53]. It is likely that reviews of medical records underestimate the rates of adverse events in prehospital care. To minimize the risk of bias, the study has been influenced by the framework formulated by Kaji et al. [54].

By using a randomized sample, we included ambulance missions at all urgency levels, with a large part of the sample comprising patients with little or no need of emergency care. These patients run a low risk of adverse events due to their small care needs. This can lead to underestimations of risks in prehospital health care.

It is our judgement that the present study method poses a risk of misappraising the rates of adverse events rather than overestimating them.

## Conclusions

There were 4.3 adverse events per 100 ambulance missions in Swedish prehospital care. The majority of adverse events originated from deviations from standard of care and incomplete documentation. There was an increase in the risk of adverse event among patients who the EMS team assessed as having a life-threatening condition (priority 1). Most adverse events were possible to avoid.

## Additional file

Additional file 1: **Table S1**. Example of factors. Description of data: Example of factors that highlight the need for further exploring how the assessment and care that is offered by health care providers before arrival in hospital influence patient safety among those who call for Emergency Medical Service. (DOCX 17 kb)

## Abbreviations

AEs: Adverse events
CI: 95% Confidence interval
ED: Emergency Department
EMS: Emergency Medical Service
EMT: Emergency Medical Technicians
IQR: InterQuartile Range
IRR: Inter-Rater Rating
OR: unadjusted Odds Ratio
RN: Registered Nurses
