# Supplementary material for: Adverse events in prehospital emergency care: a trigger tool study
Source: BMC Emerg Med. 2019 Jan 24;19:14. doi: 10.1186/s12873-019-0228-3 (PMC6345067; doi:10.1186/s12873-019-0228-3)
Supplement: Supplementary file 1 — Table S1. Example of factors. Description of data: Example of factors that highlight the need for further exploring how the assessment and care that is offered by health care providers before arrival in hospital influence patient safety among those who call for Emergency Medical Service. (DOCX 17 kb) [file 12873_2019_228_MOESM1_ESM.docx]

| Bias in clinical judgement and decision making are considered as the major threat to prehospital patient safety in a systematic review [1] |
| --- |
| The prehospital identification of sepsis patients, ranging from 6% to 34% [2-3] |
| The prehospital identification of stroke patients, are estimated to ~65% [4] |

Additional file 1: **Table S1**. Examples of factors that highlight the need for further exploring how the assessment and care that is offered by health care providers before arrival in hospital influence patient safety among those who call for Emergency Medical Service.

1. Bigham BL, Buick JE, Brooks SC, Morrison M, Shojania KG, Morrison LJ. Patient safety in emergency medical services: a systematic review of the literature. Prehosp Emerg Care. 2012;16:20-35.

2. Studnek JR, Artho MR, Garner CL, Jones AE. The impact of emergency medical services on the ED care of severe sepsis. Am J Emerg Med. 2012;30:51-6.

3. Wallgren UM, Castrén M, Svensson AEV, Kurland L. Identification of adult septic patients in the prehospital setting: a comparison of two screening tools and clinical judgment. Eur J Emerg Med. 2014;21:260-5.

4. Andersson E, Bohlin L, Herlitz J, Sundler AJ, Fekete Z, Andersson Hagiwara M. Prehospital Identification of Patients with a Final Hospital Diagnosis of Stroke. Prehosp Disaster Med. 2018:1-8.
